# A Three Monoclonal Antibody Combination Potently Neutralizes Multiple Botulinum Neurotoxin Serotype E Subtypes

**DOI:** 10.3390/toxins10030105

**Published:** 2018-03-01

**Authors:** Consuelo Garcia-Rodriguez, Ali Razai, Isin N. Geren, Jianlong Lou, Fraser Conrad, Wei-Hua Wen, Shauna Farr-Jones, Theresa J. Smith, Jennifer L. Brown, Janet C. Skerry, Leonard A. Smith, James D. Marks

**Affiliations:** 1 Zuckerberg San Francisco General Hospital and Trauma Center, Room 3C-38, Department of Anesthesia and Perioperative Care, University of California, 1001 Potrero Avenue, San Francisco, CA 94110, USA; MariaConsuelo.Garcia@ucsf.edu (C.G.-R.); ali.razai@knobbe.com (A.R.); isin.geren@medeniyet.edu.tr (I.N.G.); jianlong.lou@ucsf.edu (J.L.); fraser.conrad@ucsf.edu (F.C.); wei.wen@ucsf.edu (W.-H.W.); shauna.farr-jones@ucsf.edu (S.F.-J.); 2 Knobbe Martin, 2040 Main Street, 14th Floor, Irvine, CA 92614, USA; 3 Department of Molecular Biology and Genetics, Faculty of Science, Istanbul Medeniyet University, Unalan Mahallesi, Unalan Sokak, D100 Karayolu, Uskudar-Istanbul 34700, Turkey; 4 Molecular and Translational Sciences Division, United States Army Medical Institute of Infectious Diseases (USAMRIID), Fort Detrick, MD 21702, USA; terrys2much@comcast.net; 5 Ke’aki Technologies LLC, United States Army Medical Institute of Infectious Diseases, (USAMRIID) Fort Detrick, MD 21702, USA; jennifer.l.brown436.ctr@mail.mil (J.L.B.); janet.c.skerry.ctr@mail.mil (J.C.S.); 6 Medical Countermeasures Technology, USAMRIID, Fort Detrick, MD 21702-5011, USA; leonard.a.smith@comcast.net

**Keywords:** botulinum neurotoxin, oligoclonal antibodies, serotype E botulism, recombinant antibodies, antibody engineering, mouse neutralization assay, botulinum antitoxin

## Abstract

Human botulism is most commonly caused by botulinum neurotoxin (BoNT) serotypes A, B, and E. For this work, we sought to develop a human monoclonal antibody (mAb)-based antitoxin capable of binding and neutralizing multiple subtypes of BoNT/E. Libraries of yeast-displayed single chain Fv (scFv) antibodies were created from the heavy and light chain variable region genes of humans immunized with pentavalent-toxoid- and BoNT/E-binding scFv isolated by Fluorescence-Activated Cell Sorting (FACS). A total of 10 scFv were isolated that bound one or more BoNT/E subtypes with nanomolar-level equilibrium dissociation constants (K_D_). By diversifying the V-regions of the lead mAbs and selecting for cross-reactivity, we generated three scFv that bound all four BoNT/E subtypes tested at three non-overlapping epitopes. The scFvs were converted to IgG that had K_D_ values for the different BoNT/E subtypes ranging from 9.7 nM to 2.28 pM. An equimolar combination of the three mAbs was able to potently neutralize BoNT/E1, BoNT/E3, and BoNT/E4 in a mouse neutralization assay. The mAbs have potential utility as therapeutics and as diagnostics capable of recognizing multiple BoNT/E subtypes. A derivative of the three-antibody combination (NTM-1633) is in pre-clinical development with an investigational new drug (IND) application filing expected in 2018.

## 1. Introduction

Botulinum type E neurotoxins (BoNT/E) belong to one of at least seven immunologically distinct groups of neurotoxins (BoNT/A-G and BoNT/HA [1,2]) produced by different species of bacteria from the genus *Clostridium* [3,4]. BoNT/E is produced by *Clostridium butyricum* (subtypes E4 [5,6] and E5 [7,8]) and *Clostridium botulinum* (subtypes E1, E2, E3 [9] E6 [10], E7, E8 [11], E9 [12], E10, E11 [13], and E12 [14]). Within the twelve reported E subtypes, amino acid sequence homology ranges from 99% (subtypes 1 and 2) to 88–90% (subtype 9 versus all others) [14,15]. All BoNTs have similar secondary structures consisting of three domains: a binding domain (H_C_), a translocation domain (H_N_), and a zinc metalloprotease domain (LC) [16]. However, the arrangement of the domains in serotype E toxins is significantly different from other serotypes as identified by electron microscopy [17] and X-ray crystallography [18] (PDB:3FFZ) with the LC and H_C_ packed against each other rather than in an extended conformation [19]. BoNTs are produced as a single polypeptide. In order to reach full catalytic activity, the progenitor BoNT polypeptide is cleaved between the proteolytic domain and the translocation domain, with the two resulting peptides being linked by a disulfide bridge. Toxins produced in proteolytic *C. botulinum* strains are cleaved during processing, but in non-proteolytic strains, such as those that produce all type E and some type B toxins, trypsinization is used to cleave the LC-H_N_ and achieve full toxicity [20].

Several soluble *N*-ethylmaleimide-sensitive factor attachment protein receptor (SNARE) proteins are known to interact and produce pores through which neurotransmitters are released from the nerve terminals to muscles, provoking muscle twitches. Botulinum neurotoxins act at the presynaptic level by cleaving one or more of the SNARE complex proteins, inhibiting neurotransmitter release [21], resulting in the flaccid paralysis that characterizes botulism. Because of its unique structure, BoNT/E results in the most rapid rate of intoxication of all reported serotypes [18,22].

While the majority of type E cases are foodborne, associated with raw or preserved fish or marine mammal products [23], rare cases due to other foods have been reported [7,14,24]. In addition, cases of wound botulism in intravenous drug users [25], infant botulism [5], and adult toxicoinfections associated with subtype E4, produced in *C. butyricum* [26] have been reported. Botulism outbreaks can involve one or several individuals and can be from mild to serious, even fatal, with prolonged intensive care and mechanical ventilation required [24,25].

The current treatment for adult botulism is heptavalent (serotypes A–G) equine botulism antitoxin (BAT) [27]. BAT is immunogenic, and hypersensitivity reactions have been reported, including serum sickness and asystole [27]. BAT is a F(ab’)_2_ product with short serum half-lives (7.5–34.2 h), which eliminates its use for the prevention of botulism and limits its effectiveness as a treatment. Relapses of botulism after treatment have been noted [28]. As an alternative, human monoclonal antibody (mAb)-based antitoxins composed of three mAbs [29,30] binding non-overlapping epitopes [31] are being developed. The most advanced of these is for serotype A (NTM-1631, formerly known as XOMA 3AB), which has completed a Phase 1 clinical trial with no serious adverse effects [32].

Here, we report the generation of a panel of high affinity human BoNT/E mAbs using yeast display and Fluorescence-Activated Cell Sorting (FACS) technologies. mAbs were characterized with respect to ability to bind four BoNT/E subtypes, BoNT domain bound, and epitope overlap. Epitope mapping was compared to previously published data using cryo-EM [17] and alanine scanning for four of the mAbs [33,34]. One of the mAbs was affinity-matured. A combination of three mAbs binding four BoNT/E subtypes potently neutralized each BoNT/E subtype.

## 2. Results

### 2.1. Characterization of Monoclonal Antibodies

Yeast-displayed single chain Fv (scFv) antibody libraries were constructed from the V_H_ and Vk genes of human volunteers immunized with pentavalent BoNT toxoid (serotypes A, B, C, D, and E). The construction of these libraries has previously been reported [35,36]. Yeast surface scFv display was induced, and the libraries were sorted for three to four rounds using FACS after staining with BoNT/E3. After sorting, individual colonies were picked, induced, and evaluated for BoNT/E3 binding using flow cytometry. The genes of the scFv showing significant binding to BoNT/E3 were sequenced, resulting in the identification of 10 unique scFv (Table 1). The K_D_ values of these ten yeast-displayed scFv were measured versus BoNT/E3 by flow cytometry and ranged between 0.10 and 15.4 nM. These ten scFv were further characterized for their binding to BoNT/E1, /E2, /E3, and /E4 (Table 1). One mAb (3E2) was identified that bound all four subtypes with high affinity (K_D_ < 2 nM). Two mAbs (4E17 and 3E6) were identified that bound all four subtypes, with K_D_ values ranging from 15 nM to greater than 100 nM. Four mAbs were identified that bound three subtypes (3E4, 3E5, 4E11, and 4E16).

Seven scFv were converted to IgG consisting of the human gamma 1 constant region (Fcγ1) and the human kappa or lambda constant region as previously described for subsequent studies [31]. In the case of 4E17, a higher-affinity version previously described (4E17.1) was chosen for IgG conversion [33]. The K_D_ values of IgG for BoNT/E3 ranged from 7.24 to 0.002 nM. In most cases, improved binding was seen with K_D_ values that were 1.3- to 75-fold lower than the scFv K_D_ (Table 1). Improvement in K_D_ values upon scFv to IgG conversion has been previously reported and is partially due to the stabilization of the V_H_–Vk interface provided by the constant regions [31,36].

### 2.2. Epitope Mapping

**Figure 1:**
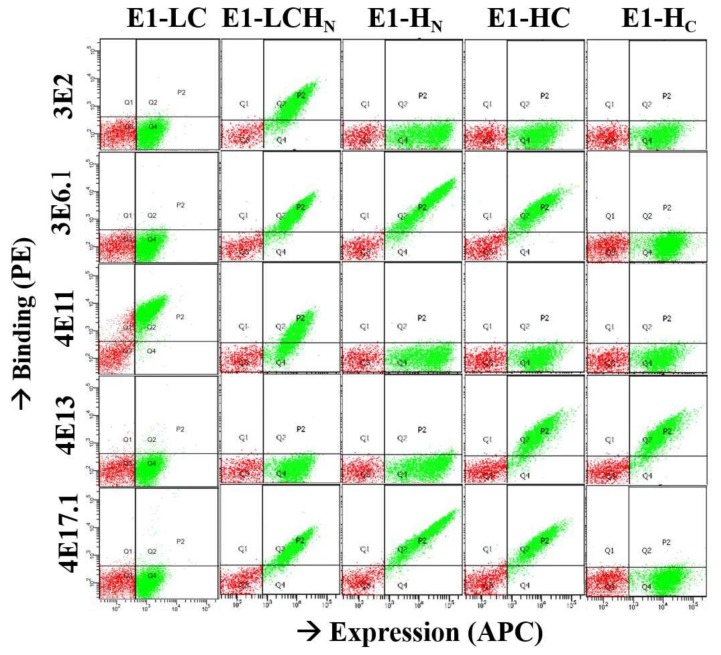
Toxin domain binding assays for five different botulinum type E neurotoxin (BoNT/E) mAbs. Yeast-displayed BoNT/E1 domains were used as antigen to detect binding for each Alexa-labeled IgG. Flow cytometry dot plots show binding results after the independent incubation of each IgG with each expressed domain: light chain (LC), proteolytic and translocation domains (LC-H_N_), translocation domain (H_N_), translocation plus binding domain (HC), and binding domain (H_C_). All domains were well-displayed as indicated by a >2 log shift with anti-SV5-Alexa-647. All domains are bound only by domain-specific mAbs, with no binding of mAbs specific to other domains, 3E2 to LC-H_N_, 3E6 and 4E17 to H_N_, 4E11 to LC, and 4E13 to H_C_.

The binding location of the different mAbs was determined using yeast display of five different BoNT/E1 constructs comprising single or combined functional domains of BoNT/E1: LC-H_N_ (proteolytic and translocation domains), H_N_ (translocation domain), HC (translocation plus binding domain), H_C_ (binding domain), and LC (light chain) as previously described for BoNT/A mAbs [37]. MAbs 3E6.1 (a higher affinity derivative of 3E6, see below) and 4E17.1 (a higher affinity derivative of 4E17) [35] bind to the H_N_ domain, while 4E13 binds to the H_C_ domain (Figure 1 and Table 1). MAb 3E2 binds the LC-H_N_, but did not bind either the H_N_ or LC alone (Figure 1), indicating that this mAb has a complex epitope spanning the LC and H_N_. MAbs 4E11 and 4E16 bind both the LC-H_N_ and LC, indicating that they bind LC epitopes.

**Figure 2:**
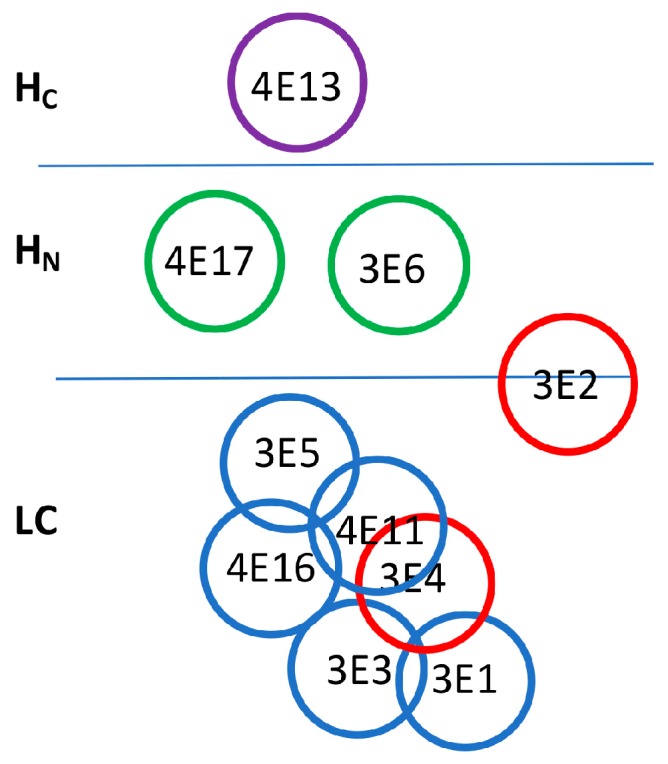
Domain epitope map of BoNT/E mAbs. The BoNT/E domain bound by each mAb was determined using yeast-displayed BoNT/E3 domains as described. Overlap of epitopes was determined using a sandwich binding assay where BoNT/E3 was captured by a yeast-displayed scFv and the ability of an individual IgG to bind BoNT/E was measured by flow cytometry (see Table 2). One mAb (4E13) binds H_C_, two mAbs (4E17 and 3E6) bind non-overlapping H_N_ epitopes, 3E2 binds an epitope on both the LC and H_N_, 3E5, 4E11, 4E16, 3E1, and 3E4 bind LC and the belt portion of H_N_, and 3E3 mAbs bind the LC, with each mAb having an epitope that overlaps at least one other mAb. Purple circle = binds H_C_; green circle = binds H_N_; red circle = binds epitope composed of H_N_ and LC amino acids; blue circles = binds LC.

For mAbs binding the same domain, epitope overlap was determined based on whether mAbs could simultaneously bind BoNT/E. Competition for binding was determined by capturing BoNT/E by one of the yeast-displayed scFv followed by probing with each of the IgG (Table 2). The IgG binding signal indicated whether epitopes overlapped. IgG did not bind BoNT/E when captured by the scFv from which it was derived, as indicated by the low mean fluorescence intensity (MFI) signal (Table 2). For the H_N_-binding mAbs 4E17.1 and 3E6/3E6.1, and the LC-H_N_-binding mAb 3E2, there was no inhibition of binding of any of these mAbs by the other mAbs, indicating that each of these mAbs bound non-overlapping epitopes (data not shown). Each of the other six LC mAbs showed overlap of epitopes by one or more of the other LC mAbs as indicated by values shown in bold in Table 2 that were less than 2.5-fold above control values. These studies allowed for the construction of an epitope map showing the number of unique (non-overlapping) or overlapping epitopes recognized by the BoNT/E mAbs (Figure 2). For the mAb pair 3E4/3E1, inhibition occurred in one direction (capture with 3E1) but only showed a reduced signal compared to probing with other mAbs with capture in the other direction (capture with 3E4.1). Thus, the 3E4 and 3E1 mAb footprints are shown as nearby but not overlapping. As illustrated in Figure 2, 3E2 bound a unique epitope spanning LC and H_N_ and was not inhibited by any of the other mAbs.

**Figure 3:**
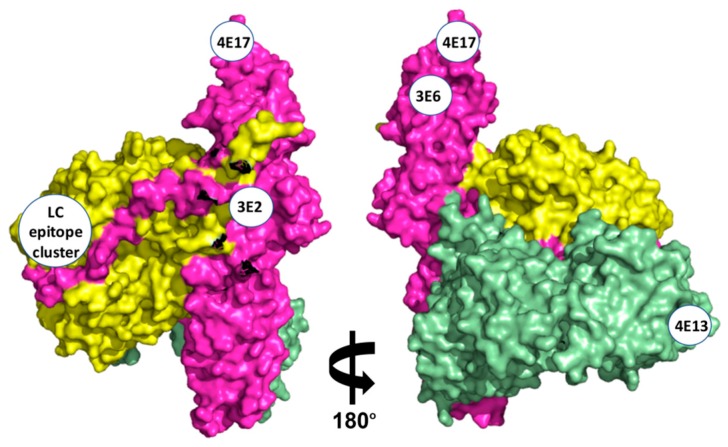
Fine epitope map of BoNT/E mAbs. Space filling representation of BoNT/E crystal structure [18], with the epitopes of mAbs 3E2, 3E6.1, 4E13, 4E16, and the LC epitope cluster and 4E17. Color-coding of the domains is: LC shown in yellow, H_N_ in magenta, and H_C_ in green.

Next, the fine epitopes of mAbs 3E1, 3E2, 3E4, 3E6.1, 4E16, 4E17.1, and 4E13 were determined by creating libraries of domain mutants and selecting for loss of binding of one of the mAbs. Putative epitopes were confirmed by introducing single alanine mutations into the mAb-specific domain and measuring the impact on affinity [37]. Epitopes were mapped onto the X-ray crystal structure of BoNT/E [18]. MAb 3E2 binds an epitope with energetically important amino acid contributions from both the LC and H_N_ at the interface where the LC packs against the H_N_ (Figure 3). This result confirms that, in solution, BoNT/E has a globular structure rather than the extended structure seen for BoNT/A (PDB: 3BTA) [19] and B (PDB: 1EPW) [38]. MAb 3E6.1 binds the H_N_ centered on alpha helix 20 at a site nearby that does not overlap with the epitope of 4E17.1 (Figure 3). Analysis of the binding of LC mAb 3E4, 4E16, 3E1, and 3E3 indicates that the LC epitope cluster is centered around amino acid 142 and consists of three non-consecutive sequence segments, mainly the tips and loops of the first, second, sixth, and seventh beta strands and the second, third, and sixth alpha helices (Figure 3). The 3E4 epitope was also identified to include amino acid side chains from the H_N_ belt as it wraps around the LC. The epitopes of mAbs 4E17.1 and 4E13 have been previously reported. MAb 4E17.1 binds an epitope at the tip of the H_N_, near the 3E6.1 epitope (Figure 3) [33] MAb 4E13 binds an H_C_ epitope consisting of amino acids R1100, R1101, K1102, S1179, Y1149, G1181, and F1184 [34]. MAb 4E13 interferes with the BoNT/E–SV2 interaction, thereby preventing binding and uptake of BoNT/E into motor neurons [34]. The general location of the epitopes of mAbs 3E6.1, 4E16, and 4E17.1 has also been confirmed by single particle electron microscopy [17].

### 2.3. Affinity Maturation of mAb 3E6

Effective BoNT neutralization requires high affinity binding (K_D_ = 1 nM or less) of three mAbs at non-overlapping epitopes that are conserved between BoNT subtypes [31]. MAbs 3E2 and 4E17.1 have non-overlapping epitopes and bind subtypes BoNT/E1, E2, E3, and E4 with high affinity (Table 3). MAb 3E6, however, has a relatively poor affinity for BoNT/E3 (1.0 × 10^−8^ M) and even poorer affinity for BoNT/E2 and E4 (Table 1). To increase the affinity of 3E6, the light chain of 3E6 was replaced with a repertoire of human light chains (chain shuffling) [33,39]. The chain-shuffled 3E6 scFv repertoire was displayed on the surface of yeast to create a library of >10^6^ transformants. The resulting yeast-displayed scFv libraries were sequentially sorted on decreasing concentrations of BoNT/E3 until the diversity had collapsed to a few unique scFv. Individual colonies were sequenced and characterized with respect to binding affinity to identify the scFv (3E6.1) with the highest affinity to BoNT/E1, E2, E3, and E4 (Table 1). MAb 3E6.1 binds BoNT/E3 with a 55-fold (2.8 × 10^−10^ M) lower K_D_ as a yeast-displayed scFv compared to 3E6 (Table 1). The 3E6.1 IgG binds BoNT/E3 with high affinity (K_D_ = 3.9 × 10^−11^ M). The binding affinity to BoNT/E4 improved, with a K_D_ of 9.7 × 10^−8^ M (Table 3), but further improvements were desired.

Since the initial round of affinity maturation involved replacing the entire light chain, the next round of affinity maturation was focused on the heavy chain variable region, specifically CDRH1. CDRH1 was chosen due to its small size (allowing for the capture of diversity in a relatively small library) and prior experience with diversifying this CDR in other affinity-maturation campaigns [33,36,40]. The affinity and cross-reactivity of 3E6.1 was further increased by diversifying five solvent-accessible amino acids (TFSSY) in the VH antigen-binding loop H1 (CDRH1) as we have previously described for affinity maturation of a BoNT/A mAb [36]. The resulting yeast-displayed scFv libraries were sequentially sorted on decreasing concentrations of BoNT/E3 until the diversity had collapsed to a few unique scFv. Individual colonies were sequenced and characterized with respect to binding affinity in order to identify the highest affinity scFv (mAb 3E6.2) to BoNT/E3. MAb 3E6.2 binds BoNT/E4 with a yeast-displayed scFv K_D_ = 1.4 × 10^−9^ M, at least 7-fold higher than the 3E6.1 scFv. The 3E6.2 IgG binds all four BoNT/E subtypes with high affinity. The K_D_ of 3E6.2 for BoNT/E4 is 5.4 × 10^−10^ M, 18-fold higher affinity than 3E6.1 (Table 3).

### 2.4. Mouse Neutralization Assays

**Figure 4:**
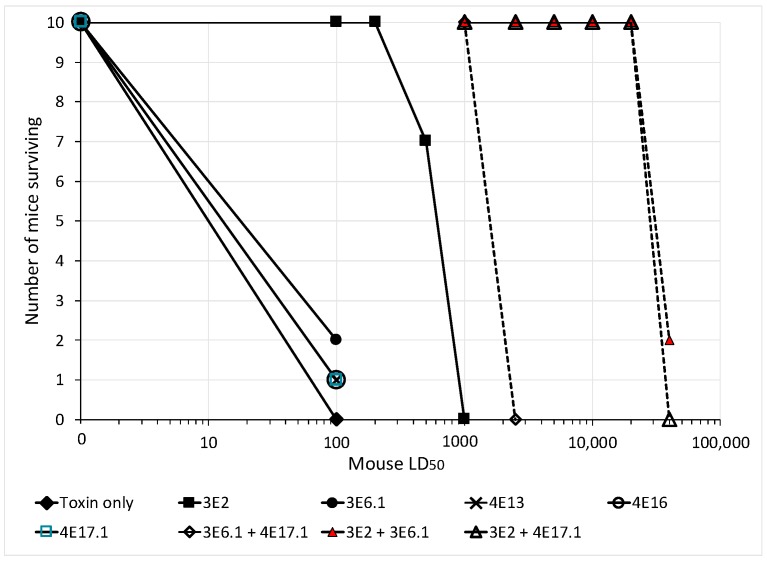
Neutralization of BoNT/E3 by single mAbs and mAb pairs as determined using the mouse neutralization assay. Fifty μg of individual mAbs or an equimolar mixture of the mAb pairs (25 μg of each mAb) was combined with the indicated amount of BoNT/E3 and injected intraperitoneally into mice. The number of mice surviving at five days was determined.

The potency of single mAbs, mAb pairs, and a combination of three mAbs was evaluated using a standard mouse neutralization assay where BoNT/E and mAbs are pre-mixed and injected intraperitoneally into mice [41]. Animals were monitored periodically and survival was determined after five days. Mice receiving 50 μg of the single mAbs 3E6.1, 4E13, 4E16, and 4E17.1 showed a prolonged time to death when challenged with 100 mouse LD_50_ of BoNT/E3; however, only one to two mice out of ten survived (Figure 4). The most protective single mAb, 3E2, showed 100% survival against 200 LD_50_ BoNT/E3 with an ED_50_ of approximately 500 LD_50_ (Figure 4). MAb pairs were significantly more potent than single mAbs. Fifty μg of an equimolar mixture of 3E6.1 and 4E17.1 completely protected mice challenged with 1000 LD_50_ BoNT/E3, which was greater than ten times more potent than either of the mAbs alone. The combination of 3E2 plus either 3E6.1 or 4E17.1 showed even greater potency, with 10/10 mice surviving challenge with 20,000 LD_50_. This is about 40 times more potent than 3E2 alone (Figure 4). The greatest potency is seen with a combination of three mAbs binding non-overlapping epitopes. Ten of ten mice dosed with 5 μg of 3E2 + 3E6.1 + 4E17.1 survived 40,000 mouse LD_50_ BoNT/E3, which is approximately 20 times more potent than the best mAb pair (Table 4). Similar high potency neutralization is seen for the mAb combination 3E2 + 3E6.1 + 4E16 (Table 4). Both of these mAb combinations neutralized BoNT/E1 with comparable potency to the BoNT/E3 neutralization (Table 4). However, only the combination of 3E2 + 3E6.1 + 4E17.1 potently neutralized BoNT/E4 (10/10 mice surviving challenge with 10,000 mouse LD_50_ plus 50 μg of mAb) (Table 4). This is not surprising given that 4E16 does not bind BoNT/E4. As expected, the combination of 3E2 + 3E6.2 + 4E17.1 was slightly more potent than the combination of 3E2 + 3E6.1 + 4E17.1 for BoNT/E4 (Table 4). 3E6.2 binds BoNT/E4 with 7-fold higher affinity than 3E6.1.

## 3. Discussion

Yeast display and sorting of scFv libraries constructed from the V-genes of humans immunized with BoNT toxoid was used to isolate a panel of ten unique human mAbs to BoNT/E. Three of the ten mAbs bound all four BoNT/E subtypes tested and two of these mAbs required affinity maturation in order to achieve our target level binding to the four BoNT/E subtypes tested (E1, E2, E3, and E4) with a K_D_ less than 1.0 × 10^−9^ M. In vivo BoNT/E3 neutralization by individual mAbs was relatively modest, as expected, with mAb 3E2 showing the most potent neutralization having an ED_50_ of approximately 500 mouse LD_50_/50 μg of mAb. The other five individual mAbs tested showed minimal protection against challenge with 100 mouse LD_50_ of BoNT/E3, including the mAb (4E13) previously shown to bind to the BoNT/E–SV2 binding site and which blocked binding and uptake of BoNT/E into motor neurons [34]. The mechanism underlying the more potent BoNT/E neutralization by mAb 3E2 compared to other mAbs is unclear; however, based on its high affinity and epitope straddling the H_N_ and LC, it could act by entering the neuron bound to BoNT and preventing or delaying intracellular translocation [42] or by preventing H_N_ dissociation from the LC-blocking substrate cleavage.

MAb combinations neutralized BoNT/E much more potently than individual mAbs, with 5.0 µg total of a triple-antibody combination able to completely neutralize 40,000 LD_50_ of BoNT/E1 or BoNT/E3 and 50 µg protecting against 10,000 LD_50_ BoNT/E4. While we did not formally titer the potency of the three-mAb combination at a higher toxin concentration, these antibody combinations could theoretically yield a neutralization capacity of 8 million mouse LD_50_/mg of mAb combination, which would be the equivalent of 8000 International Units (IU)/mg. Where we have measured the IU potency of a BoNT/A mAb combination, the value determined correlated closely with the value calculated as just described [31].

The greatly increased potency observed for the triple mAb combinations may be due to several different mechanisms. Binding of multiple mAbs could block several steps in the intoxication pathway, for example, receptor binding and translocation [42,43]. Binding of multiple mAbs also results in an avidity effect leading to an increase in mAb functional affinity [31]. It has also been shown that binding of multiple mAbs leads to rapid Fc-mediated clearance via the liver [44]. In addition, the large size of immune complexes likely slows the exit of BoNT from the circulation to its site of action.

Murine [45] camelid [46], macaque [47], and humanized macaque [47,48] mAbs neutralizing BoNT/E have been reported by others. In the case of the murine mAbs, individual mAbs were also shown to be only weakly neutralizing, with 25 μg of mAb neutralizing at most 50 LD_50_ of BoNT/E. Similar to the results reported here, 25 μg of a four or six mouse mAb combination synergized to neutralize 20,000 to 40,000 mouse LD_50_ of BoNT/E [45]. Neutralization results have been reported for individual camelid and humanized macaque mAbs (three mouse LD_50_ were neutralized by 150 mg/kg [46] and five mouse LD_50_ were neutralized by 2.5 ng of mAb [47], respectively); however, the doses of BoNT studied are so much lower than those studied here that the comparability of potency is unclear. This lack of comparability relates to the law of mass action; at a challenge dose of five LD_50_, only 80% of BoNT needs to be neutralized for 50% survival while at a challenge dose of 100 LD_50_, 99% of BoNT must be neutralized for 50% survival. In addition, the murine or macaque mAbs were only evaluated for neutralization against a single subtype.

## 4. Conclusions

The BoNT/E mAb combination described here is highly potent, with a projected dose of 0.64 mg to be equivalent to the 5100 IU of BAT in a single vial [27] (approximately 650 mg) used to treat human botulism. Component mAbs were selected based on their ability to bind and neutralize four different BoNT/E subtypes. Comparison of the fine mAb epitopes to the published sequence of the other eight BoNT/E subtypes predicts that the mAbs would bind and neutralize them, though this was not tested due to a lack of availability. Compared to equine BAT, which is immunogenic and has reported hypersensitivity reactions including cardiac arrest and serum sickness, the BoNT/E mAbs are human in sequence and should be significantly safer [27]. This work continues our efforts in developing neutralizing recombinant antitoxins to all BoNT serotypes with the goal of replacing BAT with a renewable, safer, more potent, and easier to administer recombinant antitoxin produced from stable cell lines. The most advanced of these products is XOMA 3AB, a three-mAb combination of human and humanized mAbs that has completed Phase 1 testing without serious adverse events (SAE) and with mAb serum half-lives of 12–27 days [32]. A combination of three BoNT/B mAbs has also completed Phase 1 testing in humans without SAE with pending pharmacokinetic data. The safety and high potency of mAb combinations should allow for subcutaneous or intramuscular administration compared to BAT, which must be diluted at least 10-fold into a volume of 200–500 mL and infused slowly intravenously. This makes the mAb combination much easier to administer in a mass casualty or exposure situation. Safety and a long serum half-life allows the mAb combinations to be administered before symptoms appear or to be used as an alternative to a vaccine to provide multi-month immediate immunity. The long serum half-life of two to three weeks compared to the 8–34 h half-life of BAT [27] should also prevent the recurrence of botulism post treatment that has been reported for BAT [28]. Based on the results described here, an investigational new drug application (IND) has been filed for a three-mAb combination for the treatment of botulism from type E BoNT (NTM-1633) that is expected to enter clinical studies in 2018.

## 5. Materials and Methods

### 5.1. Ethics

The USAMRIID Institutional Animal Care and Use Committee approved the animal care and use protocol to conduct the animal studies reported here. Research was conducted under an IACUC approved protocol in compliance with the Animal Welfare Act, PHS Policy, and other Federal statutes and regulations relating to animals and experiments involving animals. The facility where this research was conducted is accredited by the Association for Assessment and Accreditation of Laboratory Animal Care, International (AAALAC/I) and adheres to principles stated in the Guide for the Care and Use of Laboratory Animals, National Research Council, 2011. The specific national regulations and guidelines to which this animal care and use protocol adheres are the following: (1) 7 USC, Sections 2131–2159, Chapter 54 “Animal Welfare Act”, and (CFR, Chapter 1, Subchapter A, Parts 1–4 “Animal Welfare Regulations”; (2) Health Research Extension Act of 1985, Public Law 99-158 “Animals in Research” and the Public Health Service Policy in Humane Care and Use of Laboratory Animals; (3) Biosafety in Microbiological and Biomedical Laboratories, 5th Edition, NIH, Human and Health Services Publication (CDC) 21-112; (4) Army Regulation 40-33 “The Care and Use of Animals in DOD Research, Development, Test and Evaluation or Training Programs”; and (5) DOD Instruction 3216.01 “Use of Animals in DOD Programs”. DOD uses “The Guide for the Care and Use of Laboratory Animals”, 8th Edition, Institute for Laboratory Animal Research, National Research Council, as a guideline for the evaluation and accreditation of a program and it is based on the actual national regulations and guidelines for animal care and use programs. The animals used in this study were euthanized using carbon dioxide gas following the AVMA Guidelines on Euthanasia prior to spleen removal.

The University of California, San Francisco (UCSF) Institutional Review Board approved the human use protocol used for the studies described here. Human donors were laboratory workers being immunized to work with BoNT who were recruited via an informational letter and who signed an informed consent form.

### 5.2. Oligonucleotides for Library Construction

The primers for site-directed mutagenesis were designed and synthesized per the QuikChange Site-Directed Mutagenesis Kit (Agilent Technologies, Santa Clara, CA, USA) instructions. The primers for human and mouse library construction were synthesized as described previously [35,49]. The spiked oligonucleotide for affinity maturation of 3E6.1 was as follows:

3E6.1mutH1rev GAC CCA GTG CAT AGC 521 542 542 511 532 GAA TCC AGA GGC TGC
where:
1 = 70% A + 10% T + 10% G + 10% C
2 = 70% T + 10% A + 10% G + 10% C
3 = 70% G + 10% T + 10% A + 10% C
4 = 70% C + 10% T + 10% G + 10% A
5 = 50% G + 50% C
PYDFor1: CCCCTCAACAACTAGCAAAGGCAGCCC
PYDRev: GGTGATGGTGATGATGACCGGTACGCGTAG
Link Rev: CGACCCGCCACCGCCAGAGCCACCTCCGCC

### 5.3. Strains, Media, Antibodies, and Toxin

YPD medium was used for growth of *Saccharomyces cerevisiae* strain EBY100, SD-CAA, for selection of pYD2-transformed EBY100 and SG-CAA, and for induction of scFv expression on the surface of EBY100. *Escherichia coli* strain DH5α was used for subcloning and preparation of plasmid DNA. Pure holotoxin BoNT/E3 was purchased from Metabiologics (Madison, WI, USA). Crude extract complex for subserotypes E2 and E4 was obtained from the U.S. Army Medical Research Institute of Infectious Diseases (USAMRIID). Pure complex subserotype E1 was purchased (Wako chemicals USA Inc., Richmond, VA, USA). All IgGs were expressed from Chinese hamster ovary (CHO) cells, while the mouse anti-SV5 antibody was purified from hybridoma cells and labeled with an AlexaFluo-488 or AlexaFluo-647 labeling kit (Invitrogen, Carlsbad, CA, USA). The secondary antibody, PE-conjugated goat anti human-Fc, F(ab) was purchased (Jackson ImmunoResearch Laboratories, West Grove, PA, USA).

### 5.4. Yeast-Displayed Human scFv Library Construction and Library Sorting

To generate human mAbs that bind BoNT/E, total RNA was isolated from the blood of healthy human donors immunized with investigational pentavalent BoNT toxoid (formalin-inactivated BoNT/A, B, C, D, and E). cDNA synthesis, VH, and VK gene repertoire preparation and library construction were performed as described previously [35]. The cDNA was synthesized by RT-PCR with oligo dT as the primer using a ThermoScript RT-PCR Kit (Invitrogen, Carlsbad, CA, USA). The VH and Vk gene repertoires were amplified from cDNA by PCR with primers without gap tails and then amplified with gap-tailed primers following gel purification as previously described [33,34]. VH and Vk genes first were coupled with a (G_4_S)_3_ linker to obtain full-length scFv genes by splicing using overlap extension PCR as previously described [35,36]. The scFv gene was inserted into the NcoI/Not I restriction sites of the pYD2 plasmid and transformed into EBY100. The library size was determined by plating a serially diluted transformation mixture on SD-CAA plates. To sort the libraries, scFv display was induced by culturing in SG-CAA medium with 10% SD-CAA for at least 24 h. For sorting, the libraries were incubated with 50 nM of BoNT/E3 labeled with Alexa-647 plus SV5 mAb labeled with Alexa-488 at room temperature (RT) for 1 h. Subtype BoNT/E was used for initial sorting due to its commercial availability as pure uncomplexed holotoxin. All subsequent washing and staining steps were performed at 4 °C using ice-cold FACS buffer (phosphate-buffered saline, 0.5% bovine serum albumin, pH 7.4). After washing, yeast clones were flow-sorted on a FACSAria II, and the population with BoNT/E3-binding gated and collected. The collected yeast clones were cultured and induced for the next round of sorting. After three rounds of sorting, the collected yeast clones were plated on SD-CAA medium and cultured at 30 °C for 48 h. Individual colonies were picked, grown, and induced in 96 deep-well plates. These colonies were then screened for binding using the same staining conditions used for sorting. Unique BoNT/E3-binding clones were identified by DNA sequencing. For isolation of additional BoNT/E specific scFv, the V-genes of scFv isolated as described above (mAbs E1, E2, and E3) were converted to IgG, purified, and IgG labeled with Alexa-647. The labeling described above was then modified by incubation with unlabeled BoNT/E3 (50 nM) followed by washing and then incubation with an Alexa-647-labeled IgG (2 μg/mL) plus SV5-488. The use of labeled IgG versus labeled BoNT provided a much more robust staining and sorting reagent.

### 5.5. Measurement of K_D_ Values of Yeast-Displayed scFv

The K_D_ values of yeast-displayed scFvs were measured by flow cytometry as previously described with modification [36]. Briefly, 1 × 10^6^ yeast-displaying scFv were incubated for 1 h at room temperature in FACS buffer with six different concentrations of BoNT/E holotoxin, or complex, that spanned the range 10-fold above and 10-fold below the expected K_D_. Ice-cold FACS buffer was used to wash the samples, and 2 μg/mL each of an Alexa-647-labeled IgG that bound a different epitope was added plus SV5-488 at 4 °C and incubated for 60 min. Finally, the yeast clones were washed with ice-cold FACS buffer and the mean fluorescence intensity (MFl) of binding was measured by flow cytometry as described previously [35,50].

### 5.6. Epitope Mapping

Epitopes were classified based on the ability of mAbs to compete with each other for binding to BoNT/E as previously described [35,36]. Briefly, yeast-displayed scFv were incubated for 60 min with 25 nM of BoNT/E3. The ability of other IgG to bind to BoNT/E3 captured by the yeast-displayed scFv was detected by incubation for 60 min with 2 μg/mL of Alexa dye APC conjugated IgG and 1 μg/mL of Alexa-488-labeled SV5 antibody. Ability of the IgG to bind the scFv-captured BoNT/E was determined by flow cytometry. The IgG that bound an overlapping epitope to yeast-displayed scFv showed no APC signal, while those binding non-overlapping epitopes showed a positive APC signal.

### 5.7. Identification of mAb Domain and Fine Epitopes

To determine the BoNT/E domain bound by mAbs, synthetic genes were constructed for the BoNT/E3 H_C_, H_N_, HC, LC, and LC-H_N_. The synthetic genes were cloned into the yeast-display vector pYD2 as previously described [37] for BoNT/A domains to yield yeast-displayed H_C_, H_N_, HC, LC, and LC-H_N_ domains. Domain display was induced and individual IgG tested for binding by incubating yeast-displayed domains with 2 μg/mL IgG for 60 min at room temperature followed by incubation with 1 μg/mL of Alexa-647-labeled anti-human Fc.

To determine the fine epitopes of BoNT/E mAbs, libraries of BoNT/E3 domains LC-H_N_, H_N_, and H_C_ were prepared by introducing random mutations into the domain gene using error-prone PCR with the primers pYD-For/pYD-Rev and DNA polymerase Paq5000 (Agilent Tech, Palo Alto, CA, USA) plus 12.5 μM MnCl_2_ as previously described [37]. The PCR product was gel-purified, ligated into the NcoI/NotI restriction sites of the plasmid pYD2, and transformed into EBY100. The library was cultured in SD-CAA medium for 48 h, and then 50 mL of the culture was induced in 500 mL SG-CAA medium at 18 °C for 48 h. Amino acid residues critical for the binding of BoNT/E IgG were identified by sorting for loss of IgG binding while maintaining binding of a control BoNT/E IgG binding a non-overlapping epitope to the mAb being mapped exactly as previously described [37].

### 5.8. Site-Directed Mutagenesis

To confirm the energetically important amino acids on the BoNT/E domain critical for mAb binding, single alanine mutants of BoNT/E domains in the putative fine epitope were prepared using the QuikChange II-E Site-Directed Mutagenesis Kit (Agilent Tech, Palo Alto, CA, USA) following the manufacturer’s instructions. Briefly, primers containing the mutation were used for PCR amplification with the plasmid pYD2 containing the corresponding BoNT/E domain gene as a template for 18 cycles. The PCR product was digested by DpnI to remove the parental methylated and hemi-methylated DNA, which was then purified by StrataClean Resin and transformed into *E. coli* XL1-Blue. The alanine mutants for each gene were then individually transformed into EBY100, grown in SD-CAA medium, and induced for expression on the surface of EBY100. DNA sequencing was used to verify each construct. The ΔΔG of binding for each alanine mutant was determined by comparing the IgG K_D_ for the alanine mutant domain to the wild-type K_D_ as previously described [37].

### 5.9. Affinity Maturation Library Construction and Sorting

To increase the affinity of mAb 3E6, a V_L_ chain-shuffled scFv library was created by PCR amplifying the V_k_ gene repertoires from human immune scFv libraries using Pfu polymerase (Stratagene) and the primers LinkFor and PYDRev. To further increase V_L_ diversity, the V_L_ repertoire from a large non-immune scFv phage antibody library transferred from the phagemid vector pHEN1 and cloned into pYD2 was also used [51]. The 3E6 V_H_ DNA was amplified from the scFv gene in pYD2 using a 5′ primer that annealed upstream of the V_H_ gene (PYDFor1) and a 3′ primer that annealed in the linker gene between the V_H_ and V_L_ genes (LinkRev). The gel-purified V_H_ gene was mixed with the gel-purified V_L_ repertoires and combined with NcoI- and NotI-digested pYD2 vector DNA. This mixture was used to transform LiAc-treated EBY100 cells by three-fragment gap repair. Library size was measured as >10^7^ transformants. To select higher affinity scFvs, the V_L_-shuffled library was sorted as described above.

To create a library of CDRH1 mutants of the 3E6.1 scFv, a spiked oligonucleotide (3E6-H1Rev) was used to introduce mutations into five amino acids located in the H1 loop of the 3E6.1 scFv as previously described [40]. The oligonucleotide was designed to have a bias for a 25% or 50% wild-type amino acid at each position, depending on codon usage. Library size was 5.6 × 10^7^ transformants, and selections and new scFv validation were done as described above.

### 5.10. Measurement of Solution Phase Affinity at Equilibrium

For selected IgGs, the solution phase affinity at equilibrium and binding kinetics were measured using flow fluorimetry in a KinExA as previously described [36,50] except that BoNT/E toxins were used.

### 5.11. Measurement of In Vivo Toxin Neutralization

The mouse neutralization assay was performed as described previously [31]. Briefly, an equimolar mixture of three IgG antibodies (0.5 to 50 μg total antibody) were premixed with a range of mouse LD_50_ of BoNT/E1, /E3, or /E4 in a total volume of 0.5 mL of gelatin phosphate buffer and incubated at room temperature for 30 min. The mixture was then injected intraperitoneally into cohorts of ten mice. The animals were observed multiple times daily for clinical signs of botulism. Moribund animals were euthanized. Surviving mice at the study endpoint were tabulated.

## Figures and Tables

**Table 1:** Binding properties of BoNT/E yeast-displayed single chain Fv (scFv) and IgG. Equilibrium binding constants (K_D_) of yeast-displayed scFv for BoNT/E subtypes 1 through 4 were determined by flow cytometry. Soluble IgG K_D_ for BoNT/E3 were determined by flow fluorimetry in a KinExA. The BoNT/E domain bound by each antibody was determined as described in the Methods, and in Figure 1. 3E6.1, 3E6.2, and 4E17.1 are higher affinity derivatives of 3E6 and 4E17, respectively. 4E17 and 4E17.1 were previously reported [33].

mAb Name	Domain Bound	K_D_ (×10^−9^ M)				
		BoNT/E1, scFv	BoNTE2, scFv	BoNT/E3, scFv	BoNT/E4, scFv	BoNT/E3, IgG
3E1	LC	NB	NB	0.70	NB	ND
3E2	LC-H_N_	0.62	1.16	0.15	0.16	0.002
3E3	LC	NB	NB	0.6	NB	ND
3E4	LC-H_N_	4.7	>100	4.7	NB	0.70
3E5	LC	24.10	1.5	4.90	NB	1.4
3E6	H_N_	15.40	>100	15.4	>100	ND
3E6.1	H_N_	0.83	0.57	0.28	>10.0	0.04
3E6.2	H_N_	0.55	0.64	0.17	1.42	0.009
4E11	LC	12.67	12.39	14.20	NB	ND
4E13	H_C_	4.38	NB	9.90	NB	7.24
4E16	LC	2.22	1.43	0.20	NB	0.15
4E17	H_N_	18.37	>100	14.09	>100	ND
4E17.1	H_N_	0.16	1.27	0.1	0.60	0.24

K_D_ > 10 or 100 nM indicates that saturation was not achieved at a BoNT/E concentration of 10 nM or 100 nM, respectively; NB = no observed binding at 100 nM BoNT/E. ND = not determined.

**Table 2:** Overlap of epitopes of BoNT/E-LC-binding mAbs. The ability of each of the mAbs binding BoNT/E3 LC to inhibit the binding of other mAbs was determined by flow cytometry. Yeast-displayed scFv were used to capture BoNT/E3 followed by incubation with one IgG and binding detection by flow cytometry. Each mAb served as its own control based on the mean fluorescence intensity (MFI) value for mAb binding to BoNT captured by the same mAb. Values in bold and underlined indicate that mAb epitopes overlapped as defined by an MFI of less than 2.5-fold above the self-control background MFI (italics). 3E4.1 is a higher affinity derivative of 3E4, which was used for these studies based on its better ability to capture BoNT/E compared to 3E4.

Mean Fluorescence Intensity (MFI) of IgG Binding to Yeast scFv-Captured BoNT/E3											
Capture mAb	3E1	3E2	3E3	3E4	3E5	3E6	4E11	4E13	4E16	4E17.1	No IgG
3E1	*196*	2916	283	134	6486	1558	5357	509	1919	3742	98
3E2	2492	*276*	5503	10,394	8503	6729	10,425	5843	11,564	6870	89
3E3	143	1255	*252*	138	1966	482	2712	188	681	646	105
3E4.1	509	17,993	359	*236*	12,747	14,858	301	11,952	10,433	3262	104
3E5	2059	6026	3175	3910	*103*	1455	100	1460	104	1429	103
3E6.1	6795	18,622	13,081	17,313	7654	*230*	9309	10,982	10,185	4270	105
4E11	844	4807	802	237	139	2346	*241*	2201	210	5708	95
4E13	739	3646	1141	1334	1306	1472	1176	*238*	1976	3602	99
4E16	2093	7133	5165	9466	232	4641	101	7109	*163*	4659	85
4E17.1	8975	9039	13,085	12,820	7247	14,713	9442	6911	9667	*128*	93

**Table 3:** Binding affinity and kinetics of BoNT/E IgG for different BoNT/E subtypes. K_D_ and association rate constants for IgGs binding to different toxin subtypes determined using flow fluorimetry in KinExA. The dissociation rate constant (*k_off_*) is calculated from the K_D_ and *k_on_*.

mAb	K_D_ (×10^−12^ M)				*k_on_* (10^6^ M^−1^ s^−1^)	*k_off_* (10^−6^ s^−1^)
	BoNT/E1	BoNT/E2	BoNT/E3	BoNT/E4	BoNT/E3	BoNT/E3
3E2	11.52	15.34	2.28	17.53	1.080	2.461
3E6.1	35.11	50.93	40.32	9720	0.5272	21.26
3E6.2	6.41	7.07	8.55	542	2.136	18.27
4E16	312.13	383.12	153.64	NB	0.4444	68.28
4E17.1	730.30	204.74	239.58	492.04	0.7968	190.90

**Table 4:** Potency of combinations of three mAbs in the mouse neutralization assay. The indicated amount of BoNT and an equimolar combination of the three mAbs at the dose indicated were injected into cohorts of ten mice and the number of mice surviving was determined.

	Number of Mice Surviving/10 Mice Treated			
Antibody Dose	50 μg	10 μg	5 μg	2 μg
Antibody combination	BoNT/E1: 40,000 LD_50_			
3E2, 3E6.1, 4E16	9/10	9/10	9/10	0/10
3E2, 3E6.1, 4E17.1		10/10	10/10	0/10
	BoNT/E3: 40,000 LD_50_			
3E2, 3E6.1, 4E16		9/10	9/10	1/10
3E2, 3E6.1, 4E17.1		9/10	10/10	0/10
3E2, 3E6.2, 4E17.1		9/10	10/10	0/10
	BoNT/E4: 40,000 LD_50_			
3E2, 3E6.1, 4E16	0/10			
	BoNT/E4: 10,000 LD_50_			
3E2, 3E6.1, 4E17.1	10/10	1/10		
3E2, 3E6.2, 4E17.1	8/10	5/10		
